# Editorial: Innovative approaches to preventing and treating biofilm-associated infections

**DOI:** 10.3389/fcimb.2026.1833864

**Published:** 2026-04-15

**Authors:** Rekha Arya, Bishnu Joshi

**Affiliations:** 1 Department of Orthopedic Surgery, Arthritis and Arthroplasty Design Lab, University of Pittsburgh, Pittsburgh, PA, United States; 2 Department of Molecular Virology and Microbiology, Baylor College of Medicine, Houston, TX, United States

**Keywords:** antibacterial, antibiofilm, biofilm, biofilm-associated infections, editorial

## Introduction

**Figure 1:**
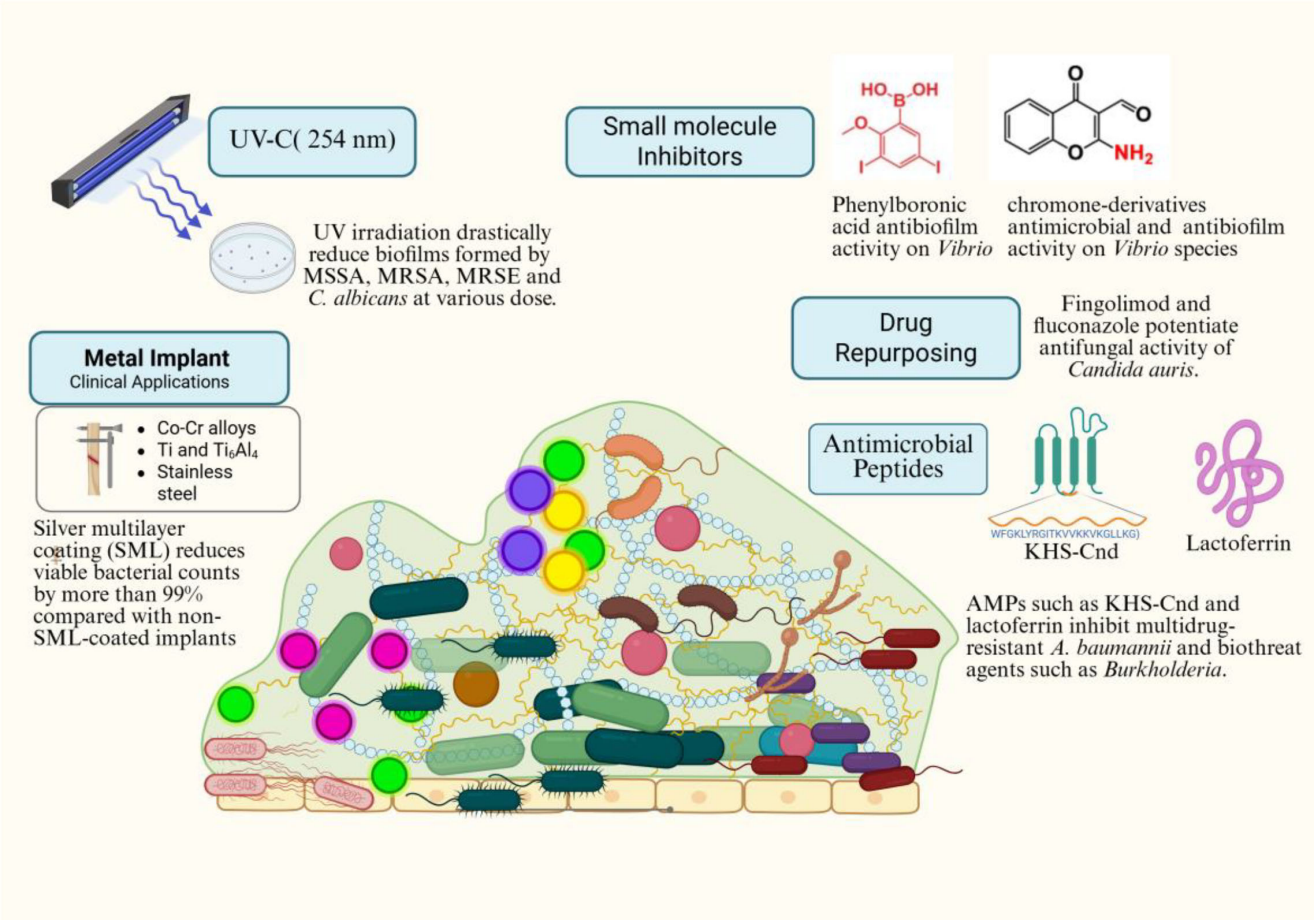
An overview of innovative approaches to prevent and treating biofilm associated infections. This figure illustrates the diverse approaches explored across 7 research articles that address the global challenge of biofilm-associated infections and antimicrobial resistance.

Biofilm-associated infections are among the most persistent and challenging issues in modern medicine, contributing to the rising incidence of hospital-acquired and surgical device-related infections. Biofilm-associated infections are inherently recalcitrant due to the complex architecture of biofilms, their persistence, and tolerance to conventional treatment, often resulting in chronic infections that are difficult to manage (Prinzi and Rohde, 2023). Biofilms allow microorganisms to survive in hostile environments, evade the immune system, and tolerate antimicrobial treatments (Nahum et al., 2025). Recently, several biofilm inhibitors and alternative strategies have been identified to prevent biofilm formation and disrupt established biofilms (Zhang et al., 2020, Blackman et al., 2021, Arya et al., 2023). The contributions in this Research Topic feature original articles that collectively explore innovative strategies targeting multidrug resistance (MDR) bacterial/and or fungal virulence, biofilm formation, and growth using peptide-based, small molecules, and protein-derived interventions. In addition, this Research Topic also highlights pharmacological repurposing of immunomodulatory therapeutics, eco-friendly decontamination strategy, and preventive biomaterial engineering approach with the overarching goal of reducing biofilm-associated infection risk (**Figure 1**).

## Small molecule-based approach: forymylchromones and halogenated phenylboronic acids

This Research Topic opens with an insightful article from Sathiyamoorthi et al., who investigated the antibacterial, antibiofilm and antivirulence properties of formylchromones, a distinct class of heterocyclic compounds, against two marine bacterial pathogens *Vibrio parahaemolyticus* and *Vibrio harveyi.* Both *Vibrio species* are responsible for foodborne gastroenteritis and disease outbreaks in aquaculture respectively, imposing substantial public health and economic burdens worldwide. Interestingly, 6-Bromo-3-formylchromone (6B3FC) and 6-chloro-3-formylchromone (6C3FC) exhibited significant inhibitory effects on bacterial growth and biofilm development in both *Vibrio* species. Their findings suggest that chromones derivatives may potentially interfere with the regulatory pathways involved in biofilm formation and cellular viability.

Complementing these findings, a second study by Sathiyamoorthi et al. from the same research group evaluates the antibacterial and antibiofilm activity of halogenated phenylboronic acid derivatives against *V. parahaemolyticus*. Among the compounds screened, 3,5-diiodo-2-methoxyphenylboronic acid (DIMPBA) and 2-fluoro-5iodophenylboronic acid (FIPBA) exhibited substantial antibacterial and antibiofilm efficacy, with both compounds inhibiting planktonic cell growth and preventing biofilm formation at a MIC of 100 µg/mL. Notably, the incorporation of halogen substituents into the phenylboronic acid scaffold significantly enhances biological activity, providing valuable structure-activity relationship insights that may guide the rational design of next-generation therapeutic agents through medicinal chemistry approaches.

By consistently demonstrating antibiofilm activity against two clinically and economically important *Vibrio* pathogens, both studies support the potential of using small-molecule interventions to target biofilm-associated infections.

## Drug repurposing: fingolimod as an adjuvant antifungal agent

The pharmacological repurposing of FDA-approved therapeutics is considered fast-track attractive approach to expand the antimicrobial arsenal, particularly in the face of escalating drug resistance as these drugs have already passed the toxicity and clinical trials (Donlin and Meyers, 2022). *Candida auris*, an emerging eukaryotic pathogen, has been classified as critical priority pathogen due to its MDR and the urgent need for new antifungal therapies. In this context, Bae et al. have investigated the potential of fingolimod, an immunomodulatory drug used to treat multiple sclerosis, in combination with the antifungal agent fluconazole against fluconazole-resistant *C. auris* (FRCA). Interestingly, the combination of fingolimod and fluconazole significantly inhibited biofilm formation, reduced fungal metabolic activity, and suppressed the expression of virulence-associated genes including *ERG11, CDR1*, and *KRE6* that mediate azole resistance, efflux pump activity, and extracellular matrix production, respectively, all play critical roles in biofilm-associated pathogenicity. These findings suggest that fingolimod may serve as a promising adjuvant therapy to enhance the efficacy of fluconazole against FRCA.

## Antimicrobial peptide and protein-derived interventions

### KHS-Cnd: an antivirulence peptide against *Acinetobacter baumannii*

Many pathogenic bacteria possess an arsenal of virulence factors, including adhesins and toxins, which enable them to colonize host tissues and establish infection. Targeting these virulence mechanisms rather than directly killing the bacteria represents an emerging paradigm in anti-infective research and antimicrobial drug development as this approach potentially minimizing resistance development and preserves collateral damage to the commensal microbe (Rasko and Sperandio, 2010). In this regard, antimicrobial peptide (AMPs) have emerged as an innovative antivirulence strategy. AMPs represent nature’s vast and untapped reservoir of promising therapeutic molecules produced by diverse organisms. Several well-characterized peptides such as LL-37, Human Defensins, Nisin, Melittin have been extensively studied for their antibacterial activities, which include membrane disruption, pore formation, inhibition of biofilm development, and immunomodulatory effects (Oliveira Júnior et al., 2025). In the Research Topic, Artini et al., reported the rational design of a chionodracine-derived peptide (KHSCnd), an antimicrobial peptide originally identified in the Antarctic icefish *Chionodraco hamatus*. The peptide was engineered by substituting histidine and serine residues with lysines to increase its positive charge. The resulting 22-mer peptide (~2.5kDa), KHS-Cnd was evaluated against MDR strains of *Acinetobacter baumannii*, a prominent member of the ESKAPE pathogens, that has been designated as a critical-priority pathogen by the WHO due to emergence and transmission of carbapenem-*resistant A. baumannii* in hospital settings. A. *baumannii*, an opportunistic bacterial pathogen, is generally associated with HAI and are intrinsically resistant to various class of antibiotics including β-lactams, fluoroquinolones and aminoglycosides as well as ability to persist on abiotic surfaces and medical devices through robust biofilm forming capacity. In the current study, KHS-Cnd demonstrated a strong ability to disrupt virulence-associated traits, particularly biofilm formation and structural integrity, without relying solely on direct bactericidal activity, however, its *in vivo* efficacy in a murine model remains to be evaluated. By targeting virulence determinants instead of essential bacterial processes, such agents may exert lower selective pressure for resistance development while also potentially improving the effectiveness of existing antimicrobial therapies.

### Lactoferrin: a bioactive host-defense protein against biothreat agents

Xander et al. broaden the thematic scope of this Research Topic by exploring the antimicrobial potential of lactoferrin, an iron-binding glycoprotein that is also essential component of host immunity. The authors demonstrate that a novel purified bioactive form of bovine lactoferrin has inhibitory effects on both bacterial growth and biofilm formation in biothreat agents, including *Francisella tularensis* (causative agent of tularemia), *Burkholderia pseudomallei* (melioidosis), and *Burkholderia mallei* (glanders). These findings highlight the therapeutic potential of naturally derived bioactive molecules and support further exploration of host-defense proteins as antimicrobial alternatives or adjuncts against high-risk bacterial biothreat agents. A novel purified bioactive form of lactoferrin is shown to exert inhibitory effects on both bacterial growth and biofilm formation. This highlights the therapeutic potential of naturally derived host-defense proteins. These findings support further investigation of endogenous antimicrobial proteins as standalone agents or adjuncts within multifaceted infection control strategies.

### Eco-friendly decontamination: UV-C irradiation against healthcare associated biofilms

Biofilm-forming microorganisms contaminating hospital surfaces and medical or surgical instruments are the major drivers of nosocomial infections and amplifiers of biofilm-associated multidrug resistance. While conventional chemical disinfectants, including alcohols, glutaraldehyde, formaldehyde, hydrogen peroxide, quaternary ammonium compounds, and sodium hypochlorite remain widely used, their known toxicity and environmental concern require other ecofriendly alternatives. Palma et al. address this gap with a systematic evaluation of UV-C irradiation at 254 nm (low-pressure mercury lamps) as a chemical-free, residue-free decontamination strategy. The authors have evaluated UV-C susceptibility across three experimental conditions: planktonic cultures, 24-hour mature biofilms, and 24-hour biofilms established on stainless steel discs against a panel of clinically relevant hospital pathogens, including Methicillin-susceptible *Staphylococcus aureus* (MSSA, ATCC 29213), methicillin resistant *S. aureus* (MRSA, ATCC 43300), *Escherichia coli* (ATCC 25922), *Pseudomonas aeruginosa* (ATCC 15442), *Candida albicans* (ATCC 14053), and a clinical methicillin-resistant *Staphylococcus epidermidis (*MRSE*).* Among Gram-negative species, a UV dose of 467.8 mJ/cm^2^ was sufficient to reduce viable *E. coli* and *P. aeruginosa* biofilm counts to below the detection limit (<1 CFU/mL). At the highest UV dose applied (946.7 mJ/cm^2^), log_10_ reductions of 4.34 ± 0.70, 4.70 ± 0.60, and 4.85 ± 0.98 were achieved against MSSA, MRSA, and MRSE biofilms, respectively. *C. albicans* emerged as the most UV-resistant organism evaluated, with a log_10_ reduction of only 3.17 ± 0.08 at the maximum dose, suggesting clinical fungal derived biofilms are harder to eliminate from UV based approach compared to bacterial associated biofilms.

### Preventive material engineering: silver multilayer coating of orthopedic implants

Implant-associated infections present significant challenges in orthopedic surgery, primarily because the biofilm formation on implant surfaces is often difficult to eliminate, necessitating additional revision surgeries or even permanent device removal. Preventing early bacterial colonization on implant materials is therefore necessary to reduce the risk of pathogen establishment and device-associated infections. Speijker et al. address this challenge through evaluation of a silver multilayer (SML) coating including titanium alloy (Ti) and cobalt-chromiummolybdenum alloy (CoCr), which are widely utilized in hip and knee prostheses. The SML coating has significant antibacterial activity against both Gram-positive (*S. aureus* and *S. epidermidis*) and Gram-negative (*P. aeruginosa*, *E. coli*). In this study, scanning electron microscopy (SEM) findings supported the CFU data by visually confirming reduced surface colonization on SML coated implants, suggesting that such coatings may help prevent prosthetic joint infections associated with biofilm formation. Further, material-dependent differences in bacterial colonization have been observed in the tested organisms, in particular, CoCr Corundum blasted (CB) surface demonstrated the lowest bacterial growth for *Pseudomonas aeruginosa*, whereas *Staphylococcus epidermidis* exhibited greater colonization on the Ti CB surface. Taken together, this study has direct clinical relevance as SML coating has a potential prophylactic strategy against early bacterial colonization and biofilm formation on orthopedic implants.

## Conclusion

The studies presented in this Research Topic collectively highlight innovative strategies to combat biofilm-associated infections using state-of-the-art molecular, biochemical, and engineering approaches. Rather than relying solely on bactericidal or fungicidal mechanisms, these investigations focus on modulating pathogenic traits, disrupting biofilm architecture, and preventing early microbial colonization, thereby limiting infection establishment and disease progression. The contributions in this Research Topic span diverse strategies, including small-molecule inhibitors, peptide-based antivirulence agents, host-defense proteins, repurposed immunomodulatory drugs, environmentally friendly decontamination technologies, and antibacterial biomaterial coatings. Together, these multidisciplinary efforts underscore the importance of integrated and multifaceted approaches to address the growing global challenge of antimicrobial resistance and biofilm-related infections. As antimicrobial resistance continues to escalate, the diversification of therapeutic and preventive strategies highlighted in this Research Topic offers significant scientific promise and translational potential for improving patient outcomes.
